# Super-resolution imaging of the neuronal cytoskeleton

**DOI:** 10.1038/s44303-024-00054-y

**Published:** 2024-12-04

**Authors:** Ciarán Butler-Hallissey, Christophe Leterrier

**Affiliations:** Aix Marseille Université, CNRS, INP UMR7051, NeuroCyto, 13005 Marseille, France

**Keywords:** Microscopy, Super-resolution microscopy, Cellular imaging, Super-resolution microscopy

## Abstract

The complexity of the brain organization and the unique architecture of neurons have motivated neuroscientists to stay at the forefront of cellular microscopy and rapidly take advantage of technical developments in this field. Among these developments, super-resolution microscopy has transformed our understanding of neurobiology by allowing us to image identified macromolecular scaffolds and complexes directly in cells. Super-resolution microscopy approaches have thus provided key insights into the organization and functions of the neuronal cytoskeleton and its unique nanostructures. These insights are the focus of our review, where we attempt to provide a panorama of super-resolution microscopy applications to the study of the neuronal cytoskeleton, delineating the progress they have made possible and the current challenges they meet.

## Introduction

The brain and associated nervous system are stunningly complex organs made of a myriad of densely packed cells that present a huge challenge to imaging at all scales. Among these cells, 86 billion neurons form the intricate network that allows for the generation, processing, and transmission of information^1^. Their extraordinary morphologies, with highly branched arborization and thousands of synaptic connections, are built, maintained, and transformed by a specialized organization of the neuronal cytoskeleton^2^. For more than a century, scientists have used a multitude of microscopy techniques to visualize and understand the architecture of the three protein polymers that form the neuronal cytoskeleton: microtubules, intermediate filaments, and actin^3,4^.

Since the beginning of the 21st century, various optical super-resolution approaches have been developed that provide a platform for the interrogation of the cellular organization at the nanoscale—below the ~220-nm lateral resolution of diffraction-limited microscopy—with the molecular specificity provided by fluorescence labeling^5–7^. Several recent reviews focus on detailing the principles of these approaches, and how to choose the one best suited for a given project^8–11^. These techniques have been readily applied to imaging the neuronal cytoskeleton and its specific challenges, such as the dense packing of scaffolds and filaments within thin processes^12–15^. Our review focuses on the insights brought by images and data obtained using super-resolution techniques in the last two decades. We will first summarize the neuronal architecture and its cytoskeletal organization, then present classic and emerging super-resolutive approaches, their principle, and their application to the visualization of the cytoskeleton in various neuronal compartments.

## Overview of the neuronal architecture and its cytoskeletal organization

**Fig. 1:**
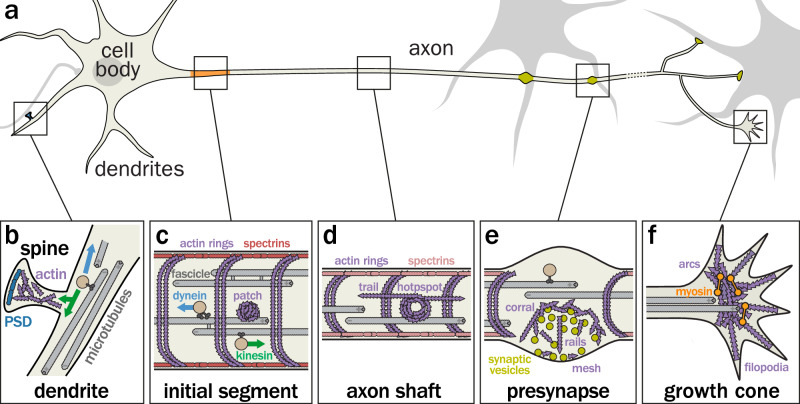
Overview of the neuronal architecture and its cytoskeletal organization. **a** Morphology of an archetypal neuron (cultured cortical neuron): the cell body and dendrites receive synaptic inputs, while the longer axon generates the action potential at its axon initial segment (orange) and contacts target cells via presynapses (green). **b** Dendrite and dendritic spine: the dendritic shaft contains longitudinal microtubules (gray) that support vesicular transport by kinesins and dynein (green and blue, respectively), while the dendritic spine is enriched in actin (purple) around the postsynaptic density (dark blue). **c** Axon initial segment: microtubules are organized in fascicles and actin forms submembrane rings spaced every 190 nm by tetramers of spectrin (red), as well as cytoplasmic actin patches. **d** Distal axon shaft: in addition to the periodic actin-spectrin scaffold, the shaft contains cytoplasmic actin hotspots and trails. **e** Presynapse: the periodic actin-spectrin scaffold is interrupted at presynapses, and specific actin nanostructures organize the presynapses by forming a corral around the vesicle reserve pool, rails, and a mesh at the active zone. **f** Growth cone: in developing neurons, the growth cone is a dynamic structure with actin-rich filopodia and actomyosin arcs (orange) that regulate the advance of microtubules.

Neurons exhibit a wide range of arborized morphologies across the different neuronal cell types^16^. Their high morphological polarization into distinct compartments reflects the asymmetric nature of information transmission in the nervous system^17,18^. A lot of what we know about the neuronal cytoskeleton comes from studying the archetypal cortical/hippocampal neurons in dissociated cultures for easy optical access. For more complex models, we look towards brain slices and living mammals, without forgetting the wealth of knowledge obtained in organisms such as fruit flies and nematode worms. Here we will briefly recapitulate the most salient features of the neuronal architecture and cytoskeletal organization, pointing to classic and recent reviews for more in-depth insights. Schematically, the neuronal cell body (also called soma) bears several dendrites that receive input from other neurons via post-synapses located along the dendritic shaft or at the tips of dendritic spines (Fig. 1a). The dendritic shaft contains longitudinal microtubules with a mixed orientation^19,20^ with peripheral, tyrosinated minus-end out and central, acetylated plus-end in microtubules^21^ traveled by motor proteins kinesin and dynein that transport cellular components^22^. Microtubules can occasionally and transiently enter dendritic spines^23,24^, which contain a high concentration of branched actin around the post-synaptic density (Fig. 1b)^25–27^.

A single axon emerges from the cell body, which transmits the action potential and signals to downstream cells via presynapses^28^. This long, thin, and arborized process is itself compartmented: at its base lies the axon initial segment, which is responsible for the generation of the action potential and the maintenance of axonal polarity (Fig. 1c)^29,30^. Axonal microtubules are uniformly oriented with their plus-ends out starting at the AIS and along the whole axon shaft^18,31^. A periodic scaffold of actin rings is regularly spaced by a layer of spectrin tetramers running along the inner side of the axonal plasma membrane (membrane-associated periodic scaffold or MPS, Fig. 1d)^32,33^. Interestingly, it is also present in thin dendrites and dendritic spine necks^34,35^, while spectrin adopts hexagonal patterns along the cell body membrane^36^. The AIS also contains a unique organization of microtubules in tight, crosslinked fascicles^37^ as well as more intracellular actin patches that help sort cargoes at the axon entrance^38,39^. The distal axon shaft also contains cytoplasmic actin hotspots and trails^40,41^ often found in the vicinity of presynaptic boutons. At these boutons, specific actin structures—corrals, mesh, and rails—play a role in organizing synaptic vesicle release and cycling (Fig. 1e)^42,43^. In developing neurons, the tip of the axon harbors a growth cone, the structure that allows for axon growth and guidance: it contains a stereotyped organization of longitudinal microtubules decorated by actomyosin arcs that can enter and stabilize thin and dynamic filopodia containing linear actin bundles (Fig. 1f)^44–46^.

## Application of the various super-resolution techniques to the study of the neuronal cytoskeleton

We will now turn to each super-resolution technique, briefly summarizing its principles and features, before detailing how it is used to visualize the neuronal cytoskeleton. Two super-resolved techniques will not be extensively featured for different reasons: first, reassignment-based super-resolution microscopy that is implemented as refinements to confocal microscopy (Zeiss Airyscan^47^, Nikon NPSARC^48^, Yokogawa SoRa^49^, VisiTech iSIM^50^, etc.) are now routinely used to push confocal microscopy to its full gain in resolution, down to ~160 nm laterally^10^. It should be noted that resolution values given throughout this review are indicative and often conservative, as the proper determination of resolution is a complex issue that involves the precision of the instrument, the density of labeling, and sample properties themselves^51^. Second, fluctuation-based super-resolution approaches (SOFI^52^, SRRF^53^, SACD^54^, etc.) allow to reach higher resolutions from a series of 10–100 consecutive exposures with minimal equipment but have seldom been used so far in neurons beyond proof of concept, such as visualizing the microtubule organization in cultured neurons^55^.

## Structured illumination microscopy (SIM)

**Fig. 2:**
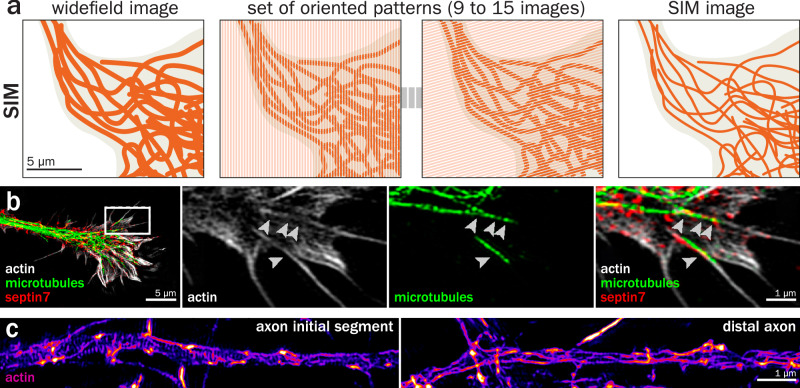
Structured illumination microscopy (SIM) of the neuronal cytoskeleton. **a** Principle of SIM: the labeled sample (microtubules in a neuron, left) is illuminated with a series of fringe patterns, varying the orientation and the phases (9–15 images total, middle). After processing, a two-fold improvement in image resolution is obtained (right). **b** Growth cone of a cultured neuron fixed and stained for actin (gray), microtubules (green), and septin7 (red), imaged by 3D SIM. Right images are zooms of the box highlighted on the left image. Arrowheads point to a septin-positive bundle between actin filaments and microtubules. Adapted from Nakos et al.^74^. Copyright 2022 National Academy of Sciences. **c** AIS (left) and distal axon (right) of cultured neurons fixed and stained for actin using phalloidin, imaged by 3D SIM. The periodic actin rings are clearly visible along the AIS but are more difficult to discern along the distal axon due to the presence of numerous longitudinal actin filaments. Adapted from Micinski et al.^224^.

We thus first turn to structured illumination microscopy (SIM), a technique whose theoretical and experimental foundations date from the early 2000s^56–59^. In its classical implementation, a SIM image is obtained by capturing a pattern of fringes modulated at the highest diffraction-limited frequency (9 for 2D SIM, 15 for 3D SIM) across the same focal plane followed by mathematical processing that exploits the Moiré effect to retrieve information down to two times the diffraction limit in both lateral and axial directions (~110 and ~250 nm, respectively, Fig. 2a)^10^. The advantages of SIM include straightforward multicolor capabilities and limited phototoxicity that makes live-cell imaging possible when compared to other super-resolution techniques. Its disadvantages are the high sensitivity to aberrations, requiring relatively thin and optically accessible samples, as well as the complexity of the mathematical post-processing that is prone to artifacts^60,61^. Several enhanced classical^62,63^ or deep learning-based^64–66^ algorithms have been developed recently that allow minimization of light exposure and reduced reconstruction artifacts.

Given its multicolor capabilities and compatibility with live-cell imaging, it is somewhat surprising that relatively few studies have used SIM to detail the organization and dynamics of the neuronal cytoskeleton. SIM has been used to delineate the morphology of dendritic spines^67,68^, but not to investigate the architecture of actin within them beyond proof of concept^69^. The limited gain in resolution offered by SIM restricts its applicability to visualize microtubules in thin processes, although it has provided beautiful images of microtubules within the cell body and proximal dendrites of osmosensory neurons in hypothalamus sections^70^. The growth cone, with its thin fan-like spread of actin-rich filopodia around a microtubule core, is well suited for SIM studies, from early demonstrations of live cell imaging^71^ to the interplay between actin bundles and vesicles in living growth cones^72^, organization or axial filopodia in fixed cells^73^ and crosstalk between actin and microtubules mediated by septins (Fig. 2b)^74^. In these examples, SIM was useful to gain better precision in assessing the spatial interplay between cytoskeletal structures.

The best-suited neuronal region for SIM might be the MPS and its actin rings separated by a layer of spectrin tetramers with a period of 190 nm, making it invisible to diffraction-limited fluorescence microscopy, but readily discernible on SIM images^33^. In fact, the first published image where the MPS is visible, showing the periodicity of spectrin-associated ankyrin G near the neuromuscular junction in Drosophila, was obtained using SIM^75^, even if the conclusive discovery of the MPS happened later thanks to localization microscopy^32^. Several studies have since taken advantage of the ease of use and high throughput of SIM to visualize cytoskeletal MPS components such as actin and spectrins along the axon^76–79^ (Fig. 2c) as well as characterize their dynamics in living cells^80–82^. Finally, while synapses have been imaged by SIM in culture^83^ or brain sections^84^, the small size of this compartment makes it difficult to resolve the intrasynaptic cytoskeletal organization, requiring more resolutive techniques such as STED or STORM.

## STimulated Emission-Depletion (STED)

**Fig. 3:**
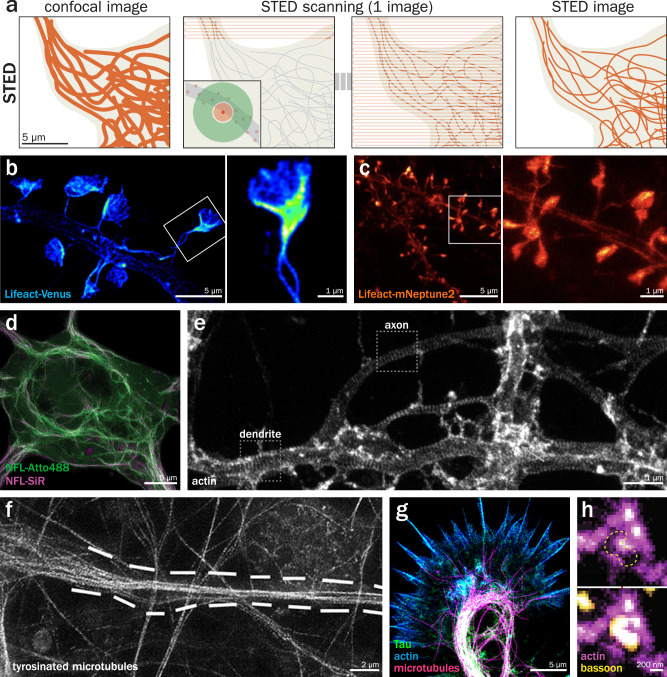
STimulated Emission Depletion (STED) microscopy of the neuronal cytoskeleton. **a** Principle of STED: on a sample labeled with suitable fluorophores (left), a double beam made of a focalized excitation spot (middle left, inset, red) superimposed with a donut-shaped depletion beam (green) excites fluorophores only in a restricted spot, that is scanned across the whole field of view (middle right), providing a higher resolution STED image (right). **b** Dendritic spines along a dendrite in a fixed rodent neuronal culture expressing the Lifeact-Venus actin probe imaged by STED. Zoom (right) corresponds to the area highlighted in the left image. Adapted from Chevy et al.^93^. **c** Dendritic spines along the dendrites of neurons expressing the Lifeact-mNeptune2 far-red actin probe imaged by 2D STED in the cortex of a living mouse. Zoom (right) corresponds to the area highlighted in the left image. Adapted from Wegner et al.^98^. **d** STED image of cultured mouse cortical neurons engineered to express the neurofilament light chain (NFL) with an unnatural aminoacid which was subsequently labeled two days apart using SiR-tetrazine (before fixation, magenta) and Atto488-tetrazin (after fixation, green). Adapted from Arsić et al.^100^. **e** 2D STED image of cultured rat hippocampal neurons fixed and stained for actin using phalloidin. Highlighted areas show a dendritic and an axonal segment with actin rings forming part of the MPS. Adapted from Lavoie-Cardinal et al.^35^. **f** STED image of cultured rat cortical neuron extracted and stained for tyrosinated tubulin (gray), showing the presence of tyrosinated microtubules within the axon initial segment (dotted lines). Adapted from Zempel et al.^116^. **g** STED image of an axonal growth cone in a culture of golden Syrian hamster neurons fixed and stained for actin (blue), microtubules (magenta), and Tau (green). Adapted from Biswas et al.^118^. **h** 2D STED image of cultured mouse hippocampal neurons stained for actin (magenta) and bassoon (yellow), showing actin surrounding the active zone (dotted yellow line) in a presynapse. Adapted from Ogunmowo et al.^123^.

STED is a technique that was conceptualized in 1994^85^ and experimentally demonstrated in 1999–2000^86^. Commercial instruments started being available at the end of the 2000s, leading STED to be more widely used to visualize cellular architecture in the following years^87^. Its principle is based on local de-excitation (through a photophysical process called stimulated emission) by a donut-shaped laser. This de-excitation donut is precisely positioned around the diffraction-limited focal spot of point-scanning excitation laser, resulting in fluorescence emission only from a sub-diffraction spot at the center of the beams (Fig. 3a). It is a scanned technique like classical confocal microscopy that has the advantage of reaching resolutions below ~50–60 nm laterally^10^ without requiring the acquisition of many single images or elaborate processing, and can readily enhance resolution axially thanks to hollow three-dimensional depletion beams. Its disadvantages are the requirement for specific stable fluorophores, and high laser intensities necessary for the depletion effect that generates bleaching and phototoxicity for living cells, although later developments try to alleviate this aspect by using photoconvertible probes (RESOLFT)^88^, modulating the depletion beam depending on the local signal (DyMIN)^89^, or leveraging deep learning-based denoising on low-power acquisitions^90^. Access to fluorescence lifetime via approaches like TauSTED allows for better signal over the background and spectral discrimination of fluorophores^91^.

One of the most successful applications of STED to the visualization of the neuronal cytoskeleton has been the imaging of dendritic spines using actin probes^92^. Several studies have imaged dendritic actin in cultured neurons with STED, revealing sub-diffractive structures otherwise invisible or reduced to nondescript blobs by diffraction-limited imaging: the nanoscale fan-like organization of actin inside spines (Fig. 3b)^93^, the presence of periodic rings along the spine neck^34^, the existence of actin patches near shaft synapses along the dendrites^94^ or the transient formation of actin clusters in the cell body of immature neurons that supply actin to the neurites^95^. Moreover, STED has been the technique of choice for in vivo imaging below the diffraction limit, with several groups demonstrating live imaging of actin within spines in brain slices^96,97^ and even in the cortex of living mice (Fig. 3c)^98^. Recent work has also used STED to resolve neurofilaments along dendrites and their activity-dependent presence in spines^99^, as well as neurofilament organization in the cell body and dendrites using unnatural amino-acid tagging (Fig. 3d)^100^.

STED has been extensively used to visualize the MPS along axons from various types of neurons and organisms^101^ as well as at the node of Ranvier^102^, revealing the role of actin ring-associated adducin and myosin in the regulation of axon diameter^103,104^, following the formation of the MPS in developing neurons^105^, localize new MPS components along the AIS^106–108^ or the axon^109,110^, and characterize the different regulation of the MPS between axons and dendrite by neuronal activity (Fig. 3e)^35^. STED was also used to visualize the MPS in living cells^111–113^ and in the axons of *C. elegans* neurons^114^. By contrast, few studies used STED to image neuronal microtubules^115^, the resolution gained using STED being often not sufficient to distinguish individual microtubules within axons. This includes tyrosinated microtubules at the AIS^116^ and microtubule/mitochondria interactions along the axon of cultured neurons^117^. Further down the axon, the growth cone cytoskeleton was imaged by STED for higher precision in describing the role of Tau in actin-microtubule crosstalk (Fig. 3e)^118^, the role of RhoA^119^ and the formin Fnm2^120^, as well as the actin waves that travel along immature axons^121^. Finally, STED images of presynapses have shown that they are devoid of the MPS^122^ and that an actin corral separates the presynapse from the surrounding axon shaft^123^.

## Single-molecule localization microscopy (SMLM)

**Fig. 4:**
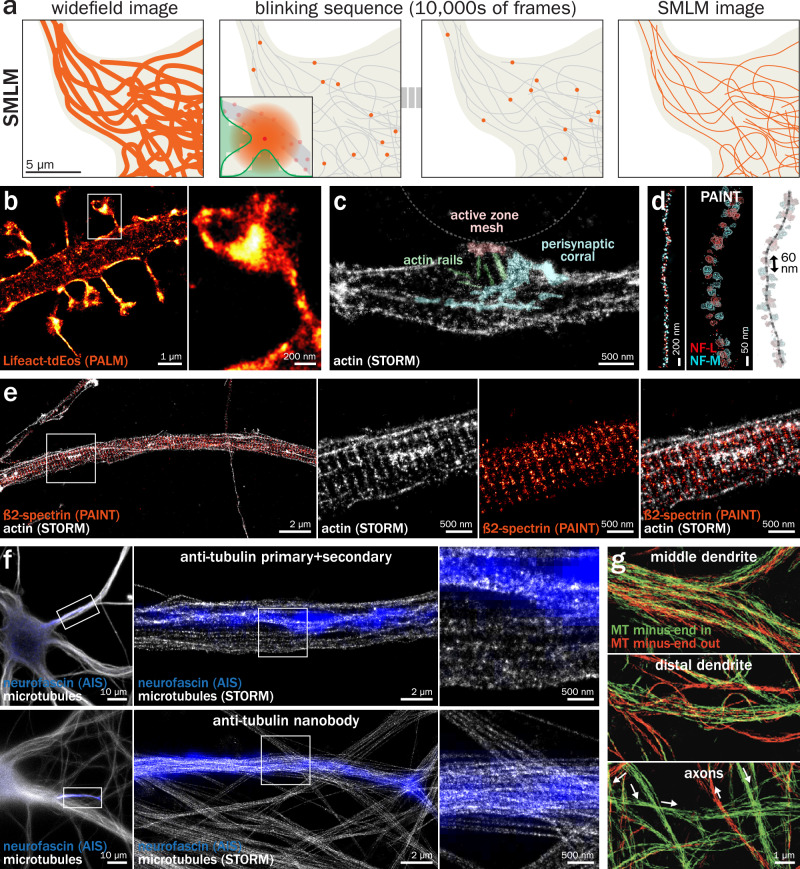
Single molecule localization microscopy (SMLM) of the neuronal cytoskeleton. **a** Principle of SMLM: a labeled sample (left) is processed so that fluorophores are sparsely and randomly blinking, and recorded for tens of thousands of frames (middle). On each frame, the PSF from each isolated blinking event is fitted to determine its center coordinates (middle left, inset). The final SMLM image (right) is made from the accumulation of blinking emitter coordinates accumulated during the whole acquisition sequence (right). **b** 2D PALM image of a dendrite in a rat hippocampal neuron in culture expressing the actin probe Lifeact fused to the photoconvertible fluorescent protein tdEos. Zoomed image (right) corresponds to the area highlighted on the left image. Adapted from Izeddin et al.^143^. **c** Image of a presynapse formed by the axon of a rat hippocampal neuron in culture on a polylysine-coated bead (dotted arc), stained for actin filaments using phalloidin and imaged by 2D STORM. The presynaptic actin nanostructures are highlighted in color: actin rails (green), mesh (red), and corral (cyan). Adapted from Bingham et al.^148^. **d** Left, 2-color 3D PAINT image of neurofilament subunits NF-L (red) and F-M (blue) along an axon of a rat hippocampal neuron in culture. The alternation of subunits is in line with the known structure of assembled neurofilaments, with a ~60 nm spacing between successive subunits (right). Adapted from Unterauer et al.^153^. **e** 2-color 2D STORM/PAINT image of the axon of a rat hippocampal neuron in culture, fixed and stained for actin with phalloidin (imaged by STORM, gray) and ß2-spectrin (images by PAINT, orange). The zoomed overlay (right, corresponding to the area highlighted on the left image) shows the alternation of the actin and ß2-spectrin bands, with a periodicity of 190 nm. Adapted from Vassilopoulos et al.^166^. **f** 2D STORM images of microtubules in the AIS of rat hippocampal neurons in culture, fixed and stained for neurofascin (blue, widefield) and microtubules (gray, STORM). Labeling with a primary anti-tubulin antibody and a fluorescent secondary antibody (top row) results in a spotty appearance that obscures the continuity of microtubules while labeling with a fluorescently tagged nanobody against tubulin (bottom row) allows for a denser staining and a more defined image that delineates individual microtubules. Unpublished data from the authors. **g** 2D Motor-PAINT images of dendrites and axons of cultured rat hippocampal neurons lightly fixed and incubated with fluorescent kinesin motors. Single-molecule localization of the moving motors allows delineation of the microtubules and their orientation (green and red) relative to the cell body. Adapted from Tas et al.^21^.

The principle of SMLM is to separate overlapping emitters in a labeled sample by inducing random, low-density blinking and precisely localizing each emission event via fitting the point spread function (PSF)^124^ and accumulating these localized emissions in a series of 1000–100,000 acquired images to create the final image (Fig. 4a)^125,126^. While the conceptual foundation of SMLM to separate fluorescent emitters closer than the diffraction limit by spreading their emission through time was proposed in 1995^127^, its experimental realization occurred more than 10 years later via two different modalities: photo-activated localization microscopy (PALM) which relies on photoactivatable (later photoconvertible) fluorescent proteins being sparsely activated and bleached (paGFP, mEos, mMaple, etc.)^128,129^, and (direct) stochastic optical reconstruction microscopy (d)STORM, where the sparse blinking of organic fluorophores from classical immunolabeling is induced by a combination of high-intensity illumination and reducing buffer that favors a transition to a stable dark state^130,131^. A third modality, DNA point accumulation in nanoscale topography (DNA-PAINT), was later developed, based on the transient binding of short fluorescent strands (imager strands) of DNA on their cognate docking strand attached to antibodies labeling the target proteins^132^. Of the three “classical” super-resolution approaches (with SIM and STED), SMLM attains the best resolution down to ~15 nm laterally and ~30 nm axially^10^ thanks to the use of engineered point spread functions such as the astigmatic PSF^126^. This resolution range allows the mapping of supramolecular complexes directly in cells and can reach structural cell biology applications^7^, which entails several disadvantages. SMLM comes at the cost of high constraints on the quality of sample preparation^133^, difficult multicolor acquisitions due to the requirement for specific blinking fluorophores (except for DNA-PAINT), challenging imaging at depths below a couple of microns, and long acquisitions (10^3^–10^6^ frames) at high excitation power (in the range of kW/cm^2^) that are largely incompatible with live-cell imaging^10^. Here again, significant efforts have been made to speed up image acquisition via faster blinking probes^134,135^, algorithms able to handle denser blinking^136–138^, and inference of a complete reconstruction from a reduced number of raw images^139,140^.

SMLM, in its various guises, has been the most widely used super-resolution technique to resolve the neuronal cytoskeleton, as the polymeric nature of the cytoskeleton allows for dense staining that is exploited to obtain the highest spatial resolution^141^. Actin and its associated proteins within dendritic spines have been imaged early on by PALM and STORM (Fig. 4b)^142–144^, and later with multicolor PAINT^145^. Spine actin has been characterized using specific segmentation algorithms in normal and Alzheimer’s disease model neurons^146,147^. Presynaptic actin, which is difficult to visualize due to the high concentration of postsynaptic actin, has recently been visualized using bead-induced presynapses and CRISPR tagging of endogenous actin by STORM and PAINT (Fig. 4c)^148^. By contrast, there are relatively few studies of the growth cone architecture using SMLM, with a couple of studies on actin^149,150^ or microtubule^151^ partners. Intermediate filaments have been visualized along axons in human tissues by 2-color STORM^152^ and characterized down to individual subunits in a recent study demonstrating highly multiplexed PAINT microscopy of up to 30 targets in cultured neurons (Fig. 4d)^153^.

The regular alternation of actin rings and spectrin tetramers that form the MPS was first discovered using STORM more than a decade ago^32^. Since then, SMLM has been the method of choice to extend and refine our knowledge of the MPS architecture and functions (Fig. 4e)^33^. STORM of actin and/or spectrin has been used to characterize the ubiquity of the MPS in various neuronal types and organisms^154^, its presence in dendrites^36^, its formation during neuronal differentiation^155^; its developmental dynamics using STORM of fixed cells and stability over minutes using live-cell PALM^156^, its alteration during degeneration^157,158^, its role in compartmenting membrane diffusion^112,159^. Multicolor PAINT has been instrumental in defining its relationship to other axonal structures, such as clathrin pits at the AIS^82^ and transport packets along the distal axon^160^. SMLM has been used to visualize the MPS at nodes of Ranvier in rats^161^ and humans^162^. Numerous components of the MPS were characterized using SMLM^163^, such as α2-spectrin^164^, adducin^32^, myosins^104,165,166^, or signaling complexes^167^. Finally, its sub-diffraction regularity makes it a sample of choice to showcase the performance of optical refinements to SMLM^168–172^, and to analysis or visualization approaches^173,174^. Beyond the MPS, STORM was also key in demonstrating the existence of other actin-base structures along axons, such as actin hotspots and trails^41,175^.

As mentioned above, resolving individual microtubules within the thin dendrites and axons is a challenge for SIM and STED. The enhanced resolution of SMLM is more adapted to this purpose, and optimized protocols have been devised to image neuronal microtubules^176,177^. STORM has been used to image microtubules at the axon entrance^178^ while PAINT was used to resolve them along axons^179,180^. However, the proximity of microtubules in AIS fascicles and thin axons is not just a resolution problem, but also a labeling one: microtubules are often too close to properly label them given the size of the primary and secondary antibodies, resulting in a spotty labeling pattern. In this case, the use of smaller nanobodies facilitates better imaging of the dense packing of microtubules (Fig. 4f)^181^. Finally, one of the most fruitful applications of SMLM to the study of neuronal microtubules has been the motor-PAINT approach, where motors traveling along microtubules of lightly fixed neurons, highlighting not only their distribution but also their orientation in dendrites and axons (Fig. 4g)^21^.

## Expansion microscopy (ExM)

**Fig. 5:**
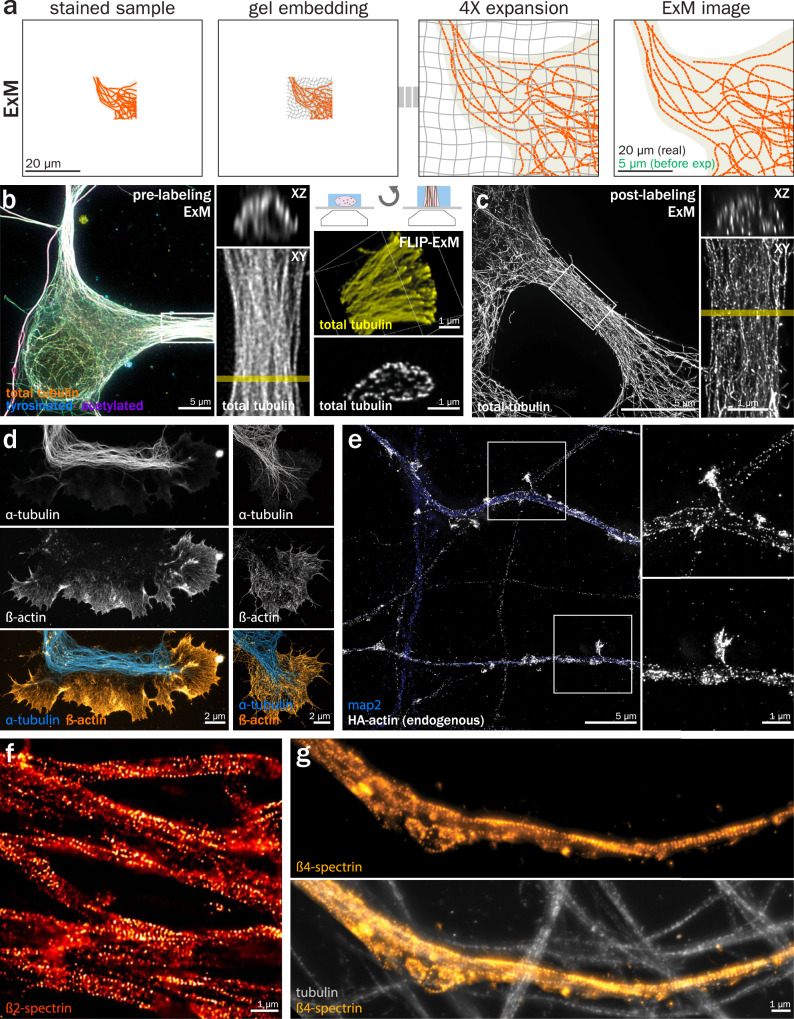
Expansion Microscopy (ExM) of the neuronal cytoskeleton. **a** Principle of ExM (case of pre-labeling ExM): a stained sample (left) is embedded in a reticulated hydrogel (middle left) before denaturation/digestion. The gel is then incubated in pure water, resulting in a ~ 4× expanded sample (64× volumetric expansion, middle right) that can be imaged by classical or super-resolved microscopy (right). **b** Maximum intensity projection of the cell body and proximal dendrites of a cultured rat hippocampal neuron pre-labeled for microtubules, expanded, imaged with 2D-STED, and deconvolved (left). A dendrite XZ cross-section is taken (top middle) at the level of the yellow line on the zoomed image (bottom middle). FLIP-ExM is used to demonstrate how reorienting ExM gel allows for a higher-resolution cross-section of dendrites (right). All scale bars on the figure are pre-expansion, i.e. corrected to consider the sample expansion factor. Adapted from Katrukha et al.^204^. **c** Maximum projection of the proximal neurite of an immature cultured rat hippocampal neuron post-labeled for microtubules, expanded, imaged on a CSU-W1 SoRa microscope, and deconvolved. A neurite XZ cross-section is taken (top left) at the level of the yellow line on the zoomed image (bottom left). Unpublished data from the authors. **d** Maximum projection deconvolved widefield (left) and confocal (right) image of the growth cone from cultured mouse hippocampal neurons that were cryofixed, expanded, and post-labeled for tubulin (cyan) and actin (orange). Adapted from Laporte et al.^207^. **e** Maximum projection deconvolved widefield image of cultured rat hippocampal neurons endogenously tagged with HA-actin that were expanded and post-labeled against the HA tag to visualize actin (gray) and Map2 (blue). Zoomed images (right) correspond to regions of actin spines highlighted on the left image. Unpublished data from the authors. **f** Maximum projection deconvolved widefield image of cultured rat dorsal root ganglia sensory neurons pre-labeled for ß2-spectrin and expanded, showing the periodic pattern of the MPS. Adapted from Martínez et al.^208^. **g** Maximum projection widefield image of the AIS of a cultured rat hippocampal neuron post-labeled for microtubules (gray, bottom) and ß4-spectrin (orange). Unpublished data from the authors.

Expansion microscopy (ExM) is a more recent technique that was first demonstrated in 2015^182^ and is still being actively developed and optimized^183^. It is largely agnostic to the complementary microscopy technique used with it, as it instead manipulates the physical properties of the sample to achieve super-resolution, which is linearly related to the expansion factor. The principle of ExM is to embed the sample within a hydrogel that crosslinks with the biological material, fragment the material by heat denaturation or proteinase digestion, and expand the hydrogen-bound fragments to visualize structures originally below the resolution limit (Fig. 5a)^184–186^. Expansion factors range from 4× and upwards depending on the protocols used, and several variants have been devised depending on the labeled target (protein, nucleic acids, lipids, etc.) and sample type^187–189^. In practice this means a microscope that has a resolution limit of 300 nm imaging a 4x expanded sample could reach a theoretical maximal “effective” resolution of 75 nm when not considering any labeling errors. Iterative expansion (i-ExM) involves successive expansion steps and has been able to reach expansion factors beyond 10× by having several expansion stages. The currently reported achievable resolution using i-ExM methods is 10–20 nm for microtubules in Toxoplasma gondii tachyzoites^190^, however the application has not been presented within the neuronal cytoskeleton. It is important to note that greater expansion factors will yield greater resolutions while sacrificing labeling density. ExM can be further divided into pre- and post-labeling: pre-labeling starts with a classical immunostained sample followed by expansion^182,191,192^, while in post-labeling ExM, the fragments are stained after expansion^193–195^. Post-labeling requires further validation of the antibodies as they need to recognize denatured proteins; the advantages to post-labeling are that it allows for better accessibility of crowded epitopes and reduces the linkage error due to primary and secondary antibodies^195^. In addition, the denser labeling and absence of fluorophore destruction by the denaturation/digestion steps results in brighter staining, which is key to mitigating the volumetric dilution of fluorescence after expansion. With the advent of ExM, its use was immediately demonstrated in brain tissue sections^182^, and it has been increasingly applied to neurobiology since^196,197^. The bulk of work in neuronal samples has been in whole organs or tissue sections to resolve diffraction-limited cell morphologies, neuronal connectomics/tracing^198–200^, and synaptic regions^201,202^.

ExM on neuronal microtubules has been one of the primary cytoskeletal structures where expansion has been applied in cultured neurons. It has been used to investigate the post-translational modification state of microtubules inside dendrites^203,204^. To counteract the poorer *Z* resolution under typical imaging conditions, the ExM gel was cut and reorientated with the neuronal cross-section facing the coverglass, thus allowing the improved counting of microtubules inside dendrites (Fig. 5b)^204^. Another approach to better-resolved microtubules is to utilize post-labeling ExM, which does not yield the resolution boost of reorienting the expanded gel but does reduce sample manipulation (Fig. 5c). Neurofilaments have also been visualized with ExM, however to date, they have been used primarily as a general marker to contextualize tissue morphology^195^ and measure expansion factors^201^.

Actin remains one of the most difficult cytoskeletal proteins to visualize with ExM because phalloidin, the gold standard to visualize filamentous actin, lacks reactive groups to link and retain it within the expanded gel. Due to this, several approaches have aimed to visualize actin with modified phalloidin and have shown some success in neuronal tissues^205,206^. Labeling of actin with antibodies has also been used after cryofixation, resulting in detailed images of microtubules and actin in growth cones^207^ (Fig. 5d). Another possibility is to tag endogenous actin with small epitope tags via CRISPR genetic engineering^148^, with labeling using anti-tag antibodies and expansion (Fig. 5e). The MPS has been difficult so far to visualize using ExM of actin, but spectrins can be readily labeled, revealing the 190-nm periodicity of the scaffold after expansion. This was done previously using pre-labeling ExM (Fig. 5f)^208^ and is also possible by post-labeling ExM (Fig. 5g).

## MINimal fluorescence photon FLUXes microscopy (MINFLUX)

**Fig. 6:**
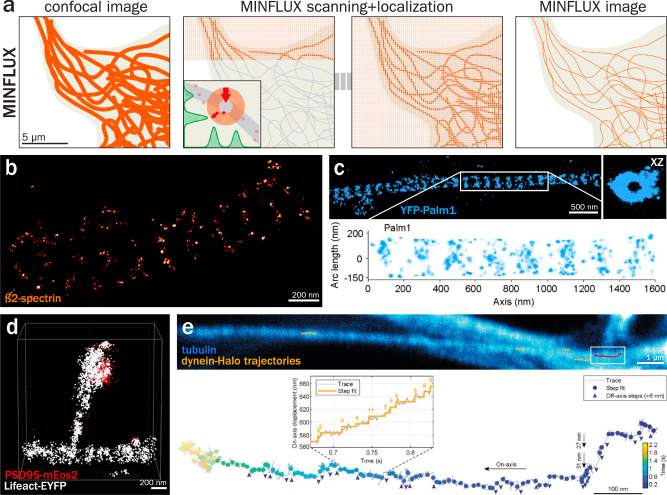
MINimal fluorescence photon FLUXes microscopy (MINFLUX) of the neuronal cytoskeleton. **a** Principle of MINFLUX: on a sample sparsely labeled with adequate fluorophores (left), a donut-shaped beam is scanned, similar to STED (middle). The fluorescence along the circular donut is monitored, and when in the vicinity of a single molecule, the beam is adjusted to place the fluorophore at its center to minimize fluorescence within the donut (middle left, inset). The resulting image is an accumulation of localized fluorophores, similar to SMLM (right). **b** Image of the axon of a mature rat hippocampal neuron in culture stained for ß2-spectrin (orange) and imaged by 2D MINFLUX. Adapted from Schmidt et al.^212^. **c** Image of the axon of a rat hippocampal neuron expressing YFP-Palm1 (paralemmin-1, blue), fixed and stained with an anti-YFP nanobody and imaged by 3D MINFLUX-PAINT. Right image shows the transverse view of the image on the left. Bottom shows the unwrapped profile of Palm1 with regular, thick bands spaced by ~190 nm. Adapted from Macarrón-Palacios et al.^109^. **d** 3D MINFLUX image of a dendritic spine within a mouse brain section expressing the postsynaptic marker PSD95-mEos2 (red) and the actin probe Lifeact-YFP (gray). Adapted from Moosmayer et al.^214^. **e** Top, confocal image of a live neuron showing microtubules (blue) and 2D MINFLUX traces (orange and red) of Halo-tag expressing endogenous dynein motors. The MINFLUX trajectory boxed on the image is magnified at the bottom, showing individual 4–11 nm steps (inset) and two large ~30 nm off-axis steps (black arrows) suggestive of dynein switching protofilament on the microtubule. Adapted from Schleske et al.^218^.

MINFLUX is a super-resolution technique that is a mix between STED and SMLM, as it uses a donut-shaped beam to image and track single fluorophores in two and three dimensions^209^. In MINFLUX, the donut beam is the excitation source with a center devoid of fluorescence excitation. This allows a single fluorophore to be precisely located by moving the beam until the fluorophore is at the center of the doughnut beam, resulting in minimal or no fluorescent photon emission (Fig. 6a)^210^. Such an approach is still susceptible to photobleaching but is not reliant on maximal photon emission to improve localization precision like SMLM. It can reach exquisite localization precision in the order of a couple of nanometers in both lateral and axial directions. This iterative single-molecule localization process means that MINFLUX is a slow technique best used on samples sparsely labeled using optimized blinking fluorophores (similar to SMLM) over small fields of view, such as nuclear pore complexes or single synapses^211^. In densely packed structures such as the neuronal cytoskeleton, MINFLUX images do not currently yield images as detailed as other super-resolution techniques. Despite this, the concept has been demonstrated for imaging the neuronal cytoskeleton, such as ß2-spectrin along the axonal MPS (Fig. 6b)^212^. More recently, MINFLUX has been used to resolve the periodic arrangement of the novel MPS component paralemmin-1 (Fig. 6c)^109^, including its relationship to adducin using a combination of MINFLUX and DNA-PAINT^213^. MINFLUX in neuronal tissue has also been recently demonstrated, visualizing actin and post-synaptic densities within dendritic spines (Fig. 6d)^214^. More methods are emerging to address the labeling density issue, for example, work by Yao et al. where they have developed a gradual labeling method with MINFLUX termed GLF-MINFLUX to visualize the microtubules and actin within the axon^215^.

Despite the current challenges of creating a “classic” microscopy image, MINFLUX has excelled as a nanoscopic tracking tool when studying motor protein movement on neuronal microtubules. The excitation donut can be set to continually and precisely follow a fluorophore during its movement. This was demonstrated within the axons of live neurons with truncated kinesin and along the dendrites of mildly fixed neurons incubated with truncated kinesin motors, resolving the 16 nm-step size of the kinesin N-terminal motor domain^216^. MINFLUX tracking has been further used to track truncated kinesin in the dendrites of immature neurons, resolving the full 16-nm steps and 8-nm sub-steps while also determining the ATP binding state based on the time between steps^217^. Tracking in live cells has also been further implemented to track CRISPR/Cas9-mediated knock-in dynein retrograde motors expressing a Halo-tag site, which was tagged just before imaging and based on the dwell time between consecutive dynein steps, they confirmed that dynein consumes a single ATP molecule per step (Fig. 6e)^218^. These studies show how MINFLUX now allows the monitoring of conformational changes of single proteins within living cells, with a temporal and spatial resolution far exceeding current FRET and fluorescence lifetime-based approaches.

## Conclusion and perspectives

With its unique nanostructures and filaments densely packed into thin processes, the neuronal cytoskeleton is the perfect target for optical super-resolution approaches. This has motivated researchers to rapidly apply every advance in super-resolution microscopy to its study^4^, providing in most cases a sharper insight into the spatial organization and relationships between cytoskeletal components, and sometimes entirely new insights such as the discovery of the actin/spectrin MPS. A couple of challenges remain, among which the accessibility of densely packed structures such as axonal microtubules or the postsynaptic density. These can be addressed by using smaller probes such as nanobodies^181^ or by post-labeling expansion microscopy^195^. At the highest resolution with methods like DNA-PAINT-based RESI^219^ or ExM-based ONE microscopy^220^ that approach precisions around one nanometer or less, issues shift from imaging to sample preparation: there is a point at which microscopy starts resolving the space between probes and the distance to their targets rather than the structure of the labeled object. To go further, approaches that reveal the whole biological material, such as panExM^221^, hold promise to help contextualize sparse emitters, like the electron density landscape is necessary to make sense of gold bead patterns in immunogold electron microscopy. Brighter and more photostable probes will also be instrumental in alleviating bleaching and phototoxicity, with a promising current focus on transiently interacting probes that make for better live-cell imaging (by replacing bleached emitters) and denser-fixed cell structural imaging^222,223^.

## Data Availability

No datasets were generated or analyzed during the current study.
